# Noninvasive Positive Pressure Ventilation in Chronic Heart Failure

**DOI:** 10.1155/2016/3915237

**Published:** 2016-11-06

**Authors:** Hao Jiang, Yi Han, Chenqi Xu, Jun Pu, Ben He

**Affiliations:** ^1^Department of Cardiovascular Diseases, Renji Hospital, Shanghai Jiao Tong University School of Medicine, Shanghai, China; ^2^Department of Geriatrics, First Affiliated Hospital of Nanjing Medical University, Nanjing 210029, China; ^3^Renal Division and Molecular Cell Laboratory for Kidney Disease, Renji Hospital, Shanghai Jiao Tong University School of Medicine, Shanghai, China

## Abstract

*Instruction and Objectives*. Noninvasive positive pressure ventilation (NPPV) alleviates sleep-disordered breathing (SDB) and it may improve cardiac function in SDB patients. Because large randomized controlled trials directly evaluating the impact of NPPV on cardiac function are lacking, we conducted a meta-analysis of published data on effectiveness of NPPV in improving cardiac function in patients with chronic heart failure regardless of SDB presence.* Methods*. Controlled trials were identified in PubMed, OVID, and EMBASE databases. Both fixed and randomized models were used in meta-analysis with primary outcomes of left ventricular ejection fraction (LVEF).* Results*. Nineteen studies were included with a total of 843 patients. Compared to standard medical treatment (SMT) plus sham-NPPV or SMT only, NPPV plus SMT was associated with improvement in LVEF (weighted mean difference 5.34, 95% CI, [3.85,6.82]; *P* < 0.00001) and plasma brain natriuretic peptide (BNP) level (weighted mean difference −117.37, 95% CI, [−227.22, −7.52]; *P* = 0.04) and no influence on overall mortality (RR 1.00, 95% CI, [0.96,1.04]; *P* = 0.95).* Conclusions*. In the present meta-analysis, use of NPPV plus SMT improved LVEF and reduced plasma BNP level but did not improve overall mortality in patients with chronic heart failure.

## 1. Introduction

 According to an estimate by the American Heart Association (AHA), 5.1 million American adults suffered from heart failure (HF) in 2014 [1]. Although survival has improved over time, 5-year mortality of HF patients remains high at about 50% [2–4]. HF also poses a large financial burden on the healthcare system amounting in 2012 to approximately 20.9 million dollars in direct medical costs in the United States [5].

Forty percent to half of patients with chronic heart failure (CHF) and impaired left ventricular function go on to develop sleep-disordered breathing (SDB), either obstructive or central sleep apnea (OSA or CSA) [6–9], both of which disrupt the normal relaxing effects of sleep on the cardiovascular system. Accumulated evidence suggested that SDB accelerates the progression of CHF. SDB induces hypoxia and hypercapnia, promotes autonomic imbalance with sympathetic activation and parasympathetic inhibition, and increases the blood pressure and left ventricular afterload, all of which are stimuli to myocardial ischemia [10–12], adverse cardiac remodeling [13–15], and left ventricular dysfunction [16, 17]. Among the modalities of noninvasive positive pressure ventilation (NPPV) used to treat SDB in CHF patients, continuous positive airway pressure (cPAP) attenuates central sleep apnea, improves nocturnal oxygenation and left ventricular systolic function, and reduces excitability of the overactivated sympathetic nervous system [18–21]. The more recent adaptive servoventilation (ASV) also alleviates SDB and it may improve cardiac function in CHF patients [22–27].

Perhaps because of limitations in sample size, incomplete data reporting, and population differences, not all studies on NPPV have yielded positive results in terms of cardiac function improvement. For instance, Pepperell et al. found no difference in change in left ventricular ejection fraction (LVEF) between ASV treated patients and controls [25]; Egea et al. found no significant improvement in 6 min walking test between the cPAP and control groups [28]; and both Ferrier et al. and Hastings et al. found that neither cPAP nor ASV significantly decreased plasma BNP concentration [29, 30].

We therefore sought to explore in a meta-analysis if adult patients with CHF would benefit from NPPV in improving cardiac function, in the form of cPAP or ASV, as compared to standard medical treatment (SMT).

## 2. Methods

### 2.1. Search Strategy and Literature Screening

A systematic literature review was undertaken on January 26th, 2015, using PubMed, OVID, and EMBASE databases. To retrieve the largest number of potentially related studies, the following terms were used individually: “noninvasive positive pressure ventilation,” “continuous positive airway pressure,” “bilevel positive airway pressure,” “adaptive servo-ventilation,” and “heart failure.” Articles were first screened by title and abstract, and reviews, meta-analyses, guidelines, letters, case reports, clinical trials in children, newborns, or postsurgical patients, and animal experiments were excluded. Three studies with full texts not written in English also were excluded.

The following criteria then were used to identify potentially suitable studies in a second screen: the trials were (a) well-designed randomized controlled trials (RCTs), quasirandomized controlled trials (qRCTs), and nonrandomized controlled trials; (b) enrolled subjects were adults older than 18 years and diagnosed with chronic heart failure, with or without sleep-disordered breathing (SDB); and (c) the intervention was noninvasive positive pressure ventilation (NPPV) in the form of continuous positive airway pressure (cPAP), adaptive servoventilation (ASV), or bilevel positive airway pressure (BiPAP), plus standard medical treatment (SMT), while the control treatment was SMT plus sham-NPPV or SMT only; and (d) left ventricular ejection fraction (LVEF) must be included in the study outcomes.

After the above-mentioned screening, the authors obtained the full text articles and read them carefully and independently. Articles meeting the following criteria were excluded: (a) follow-up period was less than 4 weeks; (b) number of study participants was less than 10; (c) crossover-design was excluded if data before washout were not reported or unavailable; (d) outcome LVEF was only reported by a descriptive conclusion (original data or processed data were not reported or available); and (e) subjects from subgroup analysis of the other clinical trials were repeatedly counted. In addition, we excluded articles including BiPAP from the final analysis because BiPAP worsens, rather than improves, central apneas [31].

### 2.2. Data Extraction and Processing

Two authors extracted data independently. Descriptive data include first author, publication year, study design, duration of study arms, duration of washout (if applicable), type of control used, HF inclusion criteria, SDB inclusion criteria, proportion of male patients, mean age, and mean BMI (if available). For outcome data, the mean together with standard deviation (SD) at baseline and end-trial time point was extracted for the NPPV and control arms. Standard error of the mean (SEM) was converted into SD. The change in mean was calculated as end-trial value minus baseline value. Variables reported in interquartile range were converted into mean using the method provided in the* Cochrane Handbook for Systematic Reviews of Interventions* (version 5.0.1) [32]. The change in SD was calculated using the formula also provided by the* Cochrane Handbook for Systematic Reviews of Interventions* (version 5.0.1) [32]. For the present meta-analysis, BNP was expressed in pg/mL using the conversion factor provided by Weber and Hamm [33] when different units were used. Events were defined as refractory heart failure and worsening heart failure if (1) events reported as refractory heart failure, or (2) events reported as heart failure worsening after NPPV application, or (3) events reported as unstable need emergency transplantation and readmission due to heart failure worsening. In particular, for outcome in crossover studies, the mean and variability in the NPPV and control arms before washout were extracted (if available) in this article.

### 2.3. Data Analysis

Two authors (Chenqi Xu and Hao Jiang) conducted the analyses using Review Manager version 5.2 (Nordic Cochrane Center). The pooled estimate of mean weight difference (MWD) or risk ratio (RR) with their 95% CI was calculated using random effect model or fixed effect model according to heterogeneity among studies. *Q* value and *I* ^2^ statistics, calculated when the number of analyzed studies exceeded three, were used as heterogeneity measures. A fixed effect model was used if there was no significant heterogeneity (*I* ^2^ < 50%); otherwise, the random effects model would be applied for meta-analysis. A forest plot was constructed based on the results of pooled analysis of the NPPV and control arms. The primary outcome of this meta-analysis was LVEF. Subgroup analysis based on the degree of LVEF and reported geographical location was performed. Moreover, sensitivity analyses were performed to identify the effect of a single trial by sequential elimination of each trial from the pool and then to assess the overall outcomes. Statistical significance was set at *P* value < 0.05. Risk of bias was evaluated carefully and tabulated with brief details by Jun Pu, M.D.

## 3. Results

### 3.1. Literature Search

**Figure 1:**
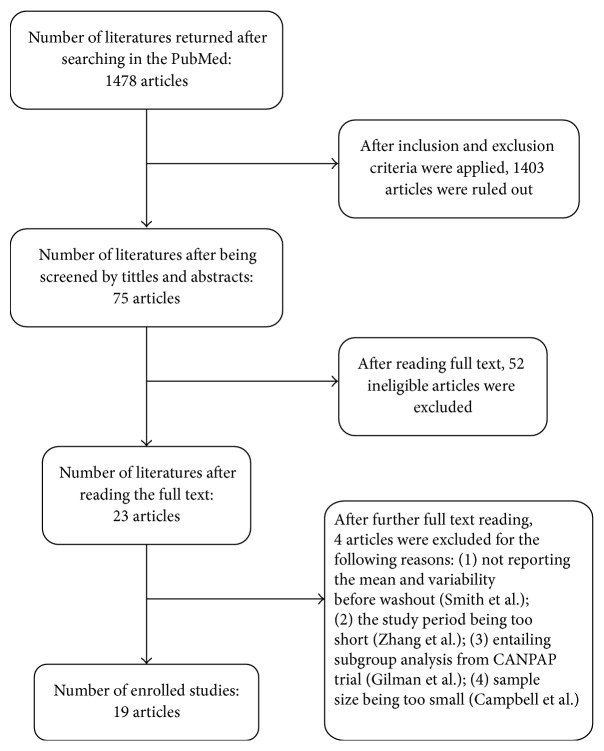
Literature screening flow.

**Figure 2:**
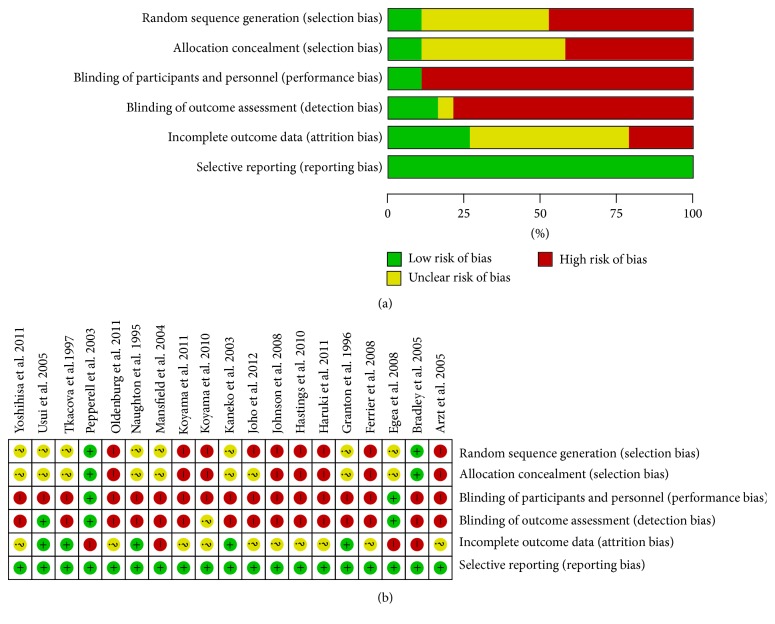
Risk of bias of the included studies. (a) Risk of bias graph; (b) risk of bias summary.

In a search of the PubMed database, 1478 potential articles were identified. After applying the prespecified exclusion and inclusion criteria, the full texts of 75 articles were read, yielding 23 eligible studies. During data extraction and analysis, 4 additional studies were excluded for different reasons: Smith et al. (2007) [34] did not report the mean and variability before washout; Zhang et al. (2006) [35] conducted a trial over a period of 1 week, which was too short to meet the inclusion criteria; Gilman et al. (2008) [36] entailed a subgroup analysis of the CANPAP trial, that is, a redundant population; and Campbell et al. (2011)'s study included a population that was not large enough. Finally, 19 studies were included in the pooled analysis. The study selection process is outlined in Figure 1. Characteristics of the included 19 studies are presented in Table 1. The risk of bias is presented in Figure 2. The included studies showed relatively high quality with an acceptable risk of bias overall (Figure 2(a)). However, there was high performance risk and detection bias in most of the studies (Figure 2(b)).

### 3.2. Meta-Analysis

#### 3.2.1. Left Ventricular Ejection Fraction (LVEF)

The weighted mean difference of the total is 5.34 favoring NPPV (95% CI, [3.85, 6.82]; *P* < 0.00001). Heterogeneity between the studies was significant (*Q* = 41.0, *P* = 0.002), and *I* ^2^ was 56%. When the study by Bradley et al. (2005) [37] was removed from the pooled analysis, *I* ^2^ changed from 56% to 33%.

The weighted mean difference of the cPAP subgroup was 3.85 favoring cPAP (95% CI, [2.28, 5.42]; *P* < 0.00001). Heterogeneity among studies in the cPAP subgroup was not significant (*Q* = 14.59, *P* = 0.15), with *I* ^2^ of 31%.

**Figure 3:**
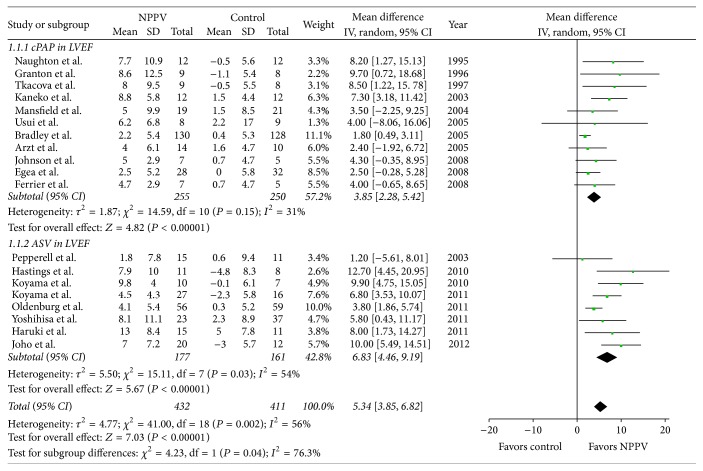
Forest plot of the effect of noninvasive positive airway pressure (cPAP and ASV) therapy for chronic heart failure on left ventricular ejection fraction (LVEF). CI: confidence interval; IV: inverse variance; SD: standard deviation; MD: mean difference.

The weighted mean difference of the ASV subgroup was 6.83 favoring ASV (95% CI, [4.46, 9.19]; *P* < 0.00001). Heterogeneity among studies in the ASV subgroup was significant (*Q* = 15.11, *P* = 0.03), with *I* ^2^ of 54% (see Figure 3).

The weighted mean difference of LVEF < 30% was 4.94 favoring NPPV (95% CI, [2.78, 7.10]; *P* < 0.00001). Heterogeneity among studies in the LVEF < 30% subgroup was significant (*Q* = 19.95, *P* = 0.006), with *I* ^2^ of 65%.

The weighted mean difference of LVEF > 30% was 5.73 favoring NPPV (95% CI, [4.03, 7.44]; *P* < 0.00001). Heterogeneity among studies in the LVEF > 30% subgroup was not significant (*Q* = 12.39, *P* = 0.26), with *I* ^2^ of 19%.

The weighted mean difference of the European subgroup was 5.05 favoring ASV (95% CI, [0.07,10.03]; *P* = 0.05). Heterogeneity among studies in the ASV subgroup was significant (*Q* = 4.96, *P* = 0.08), with *I* ^2^ of 60%.

The weighted mean difference of the Asian subgroup was 7.92 favoring ASV (95% CI, [5.58,9.96]; *P* = 0.05). Heterogeneity among studies in the ASV subgroup was not significant (*Q* = 2.43, *P* < 0.00001), with *I* ^2^ of 8.7%.

#### 3.2.2. Left Ventricular End-Diastolic Dimension (LVEDD)

Five studies reported data on change in LVEDD between before and after intervention. The weighted mean difference of the total was −1.91 favoring NPPV (95% CI, [−4.60, 0.78]; *P* = 0.16). Heterogeneity among studies was not significant (*Q* = 8.13, *P* = 0.09).

The weighted mean difference of the cPAP subgroup was 0.45 favoring control (95% CI, [−6.0, 6.89]; *P* = 0.89). Heterogeneity among studies in the cPAP subgroup was significant (*Q* = 3.79, *P* = 0.05), with *I* ^2^ of 74%.

The weighted mean difference of the ASV subgroup was −3.60 favoring ASV (95% CI, [−5.19, − 1.50]; *P* = 0.0008). Heterogeneity among studies in the ASV subgroup was small (*Q* = 1.39, *P* = 0.5), with *I* ^2^ of 0%.

#### 3.2.3. Plasma BNP Level

Six studies reported data on plasma BNP level before and after intervention; 5 from the ASV subgroup and one from the cPAP subgroup. The weighted mean difference of the total was −117.37 favoring NPPV (95% CI, [−227.22, − 7.52]; *P* = 0.04). Heterogeneity among studies was significant (*Q* = 26.40, *P* < 0.0001), with *I* ^2^ of 81%.

The mean difference of the cPAP subgroup was 4.50, not significantly favoring the control (95% CI, [−77.12, 86.12]; *P* = 0.91). And the weighted mean difference of the ASV subgroup was −152.58 favoring ASV (95% CI, [−295.81, − 9.35]; *P* = 0.04). Heterogeneity among studies in the ASV subgroup was significant (*Q* = 26.40, *P* = 0.0001), with *I* ^2^ of 83%.

#### 3.2.4. Overall Mortality

**Figure 4:**
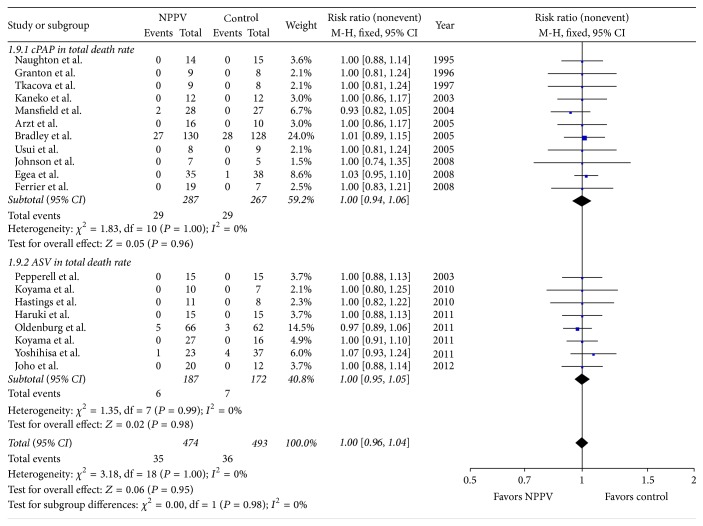
Forest plot of the effect of noninvasive positive airway pressure (cPAP and ASV) therapy for chronic heart failure on total mortality. CI: confidence interval; M-H: inverse variance; RR: risk ratio.

In 19 trials involving 913 patients, we did not find difference in overall mortality between patients treated with NPPV plus standard medical treatment (SMT) and with SMT alone (RR 1.00, 95% CI, [0.96, 1.04]; *P* = 0.95) (Figure 4).

#### 3.2.5. Adverse Events

Refractory heart failure and worsening heart failure: of the 19 studies included, 6 reported the events as defined. We found no difference in the incidence of refractory heart failure and worsening heart failure between patients treated with NPPV plus SMT and SMT alone (RR 1.07, 95% CI, [0.95,1.21]; *P* = 0.25).

Cardiac arrest: 3 studies reported the incidence of cardiac arrest and we found no difference in the incidence of cardiac arrest between the two groups (RR 1.02, 95% CI, [0.93, 1.12]; *P* = 0.63).

Angina and acute myocardial infarction (AMI): 3 studies reported the incidence of angina and AMI. We found no difference in the incidence of angina and AMI between the two groups (RR 1.01, 95% CI, [0.95, 1.08]; *P* = 0.64).

#### 3.2.6. Sensitivity Analysis

Sensitivity analyses by sequentially dropping individual trials and then evaluating the overall outcomes failed to identify any of the individual trials as having influenced the primary outcomes of the present meta-analysis to a significant extent (Table 2).

## 4. Discussion

Two main conclusions can be drawn for the present meta-analysis. Firstly, NPPV plus standard medical treatment (SMT) improved LVEF but did not improve overall mortality. Secondly, relative to SMT plus sham-NPPV/SMT alone, NPPV improved plasma BNP level but did not improve LVEDD and decrease threats of cardiac arrest events, angina, and AMI events.

### 4.1. Primary Outcomes

#### 4.1.1. NPPV Improves LVEF in Chronic Heart Failure Patients

The present meta-analysis revealed that NPPV improves cardiac function by increasing LVEF. Among included studies, the majority of patients already had reduced LVEF or were in the course of developing heart failure with reduced LVEF and therefore were considered more likely to benefit from the use of NPPV. The results are consistent with those of many previous studies [19, 20, 30, 37–45]. However, several studies [21, 25, 28, 29, 46–48], most nonrandomized and with small sample sizes, showed that NPPV had no significant effects on the improvement of the cardiac function.

In our analyses, the weighted mean difference of the cPAP subgroup was 3.85 favoring cPAP, while that of the ASV subgroup was 6.83 favoring ASV. This might indicate that ASV is better than cPAP in the improvement of LVEF. However, the conclusion did not come from the direct comparison of cPAP and ASV since none of the included studies presented such a direct comparison. Interestingly, two randomized controlled trials showed that CHF patients with SDB might gain greater benefit from treatment with ASV than with CPAP [26, 49], which was consistent with our results.

**Figure 5:**
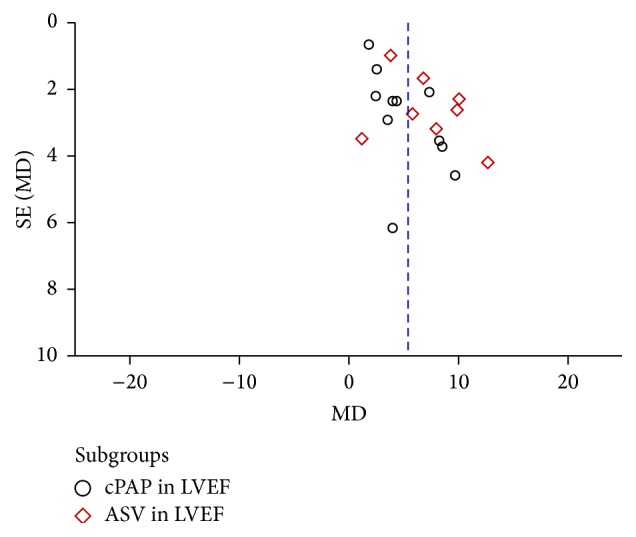
Funnel plot of NPPV on LVEF.

The total heterogeneity of the aforementioned part of the analysis is significant (Figure 5). In the cPAP subgroup, the subtotal heterogeneity is not significant (*I* ^2^ = 31%), while that of ASV subgroup is significant (*I* ^2^ = 54%). To gain further insight into the heterogeneity, we performed subgroup analyses. Firstly, we used LVEF < 30% and LVEF > 30% as grouping criteria. Then, we found that when the mean LVEF of the NPPV group before intervention exceeded 30%, the weighted mean difference was favoring NPPV and the heterogeneity was small. However, when the mean LVEF of the NPPV group before intervention was 30% or less, the heterogeneity was significant. The latter result probably indicated a worse status among enrolled subjects with an LVEF of 30% or less, in turn leading to a worse prognosis and underlying a statistically significant heterogeneity.

Secondly, we analyzed the difference in LVEF-change among study regions for the ASV subgroup. According to the reported geographical location where the study was conducted, we divided the 8 studies into the European subgroup (3 studies, 2 in UK, and 1 in Spain) and Asian subgroup (5 studies, all in Japan). We found that the Asian subgroup's heterogeneity was small (*Q* = 2.43, *I* ^2^ = 0%), while the heterogeneity of the European subgroup was significant (*Q* = 4.96, *I* ^2^ = 60%). The regional disparity, the difference between medical care systems, and even the different races of patients may underlie the observations in the present study, which warrant further study.

#### 4.1.2. NPPV Did Not Improve Mortality

**Figure 6:**
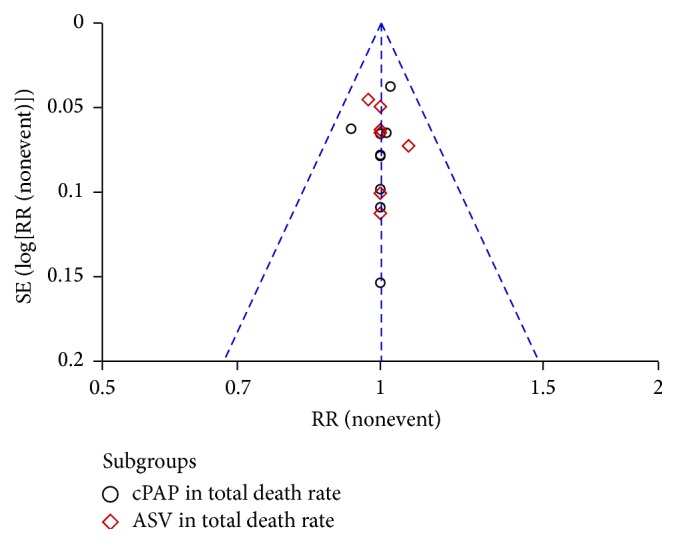
Funnel plot of NPPV on total mortality.

According to the result of the analysis, the use of NPPV plus SMT did not improve overall mortality among patients with chronic heart failure. The analysis showed good homogeneity among all 19 studies enrolled (*Q* = 3.18, *I* ^2^ = 0%) (Figure 6). Moreover, NPPV did not decrease cardiac adverse events in patients with chronic heart failure according to analysis of adverse events (including refractory heart failure and worsening heart failure, cardiac arrest, and angina and acute myocardial infarction), with good homogeneity among all these analyses.

Despite the aforementioned results, the impact of NPPV on overall mortality and cardiac adverse events remains to be further investigated. The longest follow-up period among the 19 studies was only 12 months and the shortest was 4 weeks. In a single center cohort study in Canada, patients with OSA were followed up for a decade; however, the study unfortunately did not provide information on cPAP use [50]. In a recent study (which was not included in the present analysis because of the lack of LVEF data), ASV increased the overall and cardiovascular mortality in CHF patients with OSA [51]. Thus, more studies are warranted to evaluate the effect of NPPV on 3-year, 5-year, and even 10-year mortality rate.

### 4.2. Other Secondary Outcomes

#### 4.2.1. NPPV Did Not Reduce LVEDD, but Results of Subgroup Analyses Differed

Five studies reported changes in LVEDD. The present analysis showed that NPPV did not reduce LVEDD; however, heterogeneity was significant. Subgroup analysis, however, yielded a different result. The weighted mean difference was 0.45 favoring (not significantly) control (*P* = 0.89) in the cPAP subgroup, while it was −3.60 favoring ASV (*P* = 0.0008) in the ASV subgroup, indicating that ASV might do better in reducing LVEDD than cPAP, although there was no direct comparison between cPAP and ASV. Further studies are warranted on LVEDD according to NPPV modality.

#### 4.2.2. NPPV Reduces Plasma BNP Level in Patients with Chronic Heart Failure

Among included studies, 6 reported the plasma BNP level at baseline and after intervention. One study focuses on cPAP, while the other five studies focus on ASV. The present analysis showed that the use of NPPV reduced plasma BNP level in CHF patients. Because BNP level can be used to indicate prognosis and predict mortality and clinical outcome of patients with chronic heart failure [33, 52], the reduced BNP level may indicate a better prognosis. However, the subgroup analysis showed no significant difference between cPAP and SMT in influencing plasma BNP level. Since there is only one cPAP study involved, the conclusion may be not applicable to cPAP. Conversely, ASV showed effectiveness in reducing BNP level, possibly indicating that ASV might improve the clinical outcome of CHF patients and reduce mortality. However, the heterogeneity of the analysis was very significant, and further studies are therefore warranted.

ASV was designed to meet the patients' ventilation support by providing inspiratory positive airway pressure (IPAP) and adjust the rate of change of airflow through sensing the patient airflow. cPAP, however, provided a continuous pressure which could not be adjusted according to the patients breath [53]. Several studies showed ASV was associated with significantly better compliance when compared with cPAP [49]. Interestingly, ASV was found to increase 1-year survival rate and reduce cardiovascular events in CHF patient, while cPAP did not show survival benefit among patients with CSA [37].

### 4.3. Study Limitations

Our study has several potential limitations. First, the sample sizes of component trials included in our analysis are generally not large, which may bring “small-study effects.” “Small-study effects” refer to the fact that trials with limited sample sizes are more likely to report larger beneficial effects than large trials [54, 55]. Thus, we performed sensitivity analyses to test the impact of individual trials on the overall result of meta-analysis. Second, only two studies included in our meta-analysis presented the data on NPPV compliance. Ferrier et al. pointed out patients using CPAP (>1 h per night CPAP) had the greatest increase in LVEF [29]; Joho et al. found that the change in average use of ASV correlated with changes in LVEF [41]. However, the definition of NPPV compliance in those reports was not consistent and the influence of compliance to treatment was not quantified. Thus, we did not report the influence of NPPV compliance on studied variables in the present study.

## 5. Conclusions

In the present meta-analysis, relative to SMT plus sham-NPPV/SMT alone, NPPV plus SMT improved LVEF and reduced plasma BNP level but did not improve overall mortality and adverse event rates.

## Figures and Tables

**Table 1:** Characteristics of 19 studies included in meta-analysis.

Study	Length of follow-up	Location	Ventilator mode	Ventilator parameters	Ventilator connection method	Patients	Control	Trial *n* (m/f), age (ys)	Control *n* (m/f), age	Results
Arzt et al. 2005	3 months	Germany	CPAP	CPAP: 8 to 12 cm H_2_O	Face mask	CHF with CSA	Nasal oxygen treatment	14 (NP), 64.0 ± 2	10 (NP), 65.0 ± 2	Ventilatory efficiency LVEF
Bradley et al. 2005	2 years	Canada	CPAP	CPAP: 10 cm H_2_O	Face mask	CHF with CSA	SMT	128 (125/3), 63.2 ± 9.1	130 (123/7), 63.5 ± 9.8	Effect of CPAP on CSA and LVEF Death rates
Egea et al. 2008	3 months	Spain	CPAP	NP	Face mask	CHF with SA	Sham-CPAP	28 (24/4), 64.0 ± 0.9	32 (29/3), 63.0 ± 1.6	AHI and LVEF
Ferrier et al. 2008	6 months	New Zealand	CPAP	NP	Face mask	CHF with OSA	SMT	19 (16/3), 58.5 ± 11.2	7 (3/4), 60.3 ± 4.3	LVEF, SBP, BNP, LVESD, LVEDD
Granton et al. 1996	3 months	Canada	NCPAP	CPAP: 10 to 12.5 cm H_2_O	Nasal mask	CHF with CSR-CSA	SMT	9 (NP), 58.3 ± 2.2	8 (NP), 58.0 ± 2.0	MIP and MEP, LVEF, dyspnea
Haruki et al. 2011	6 months	Japan	ASV	EPAP: 5 cm H_2_O IPAP: 3–10 cm H_2_O	Face mask	CHF	SMT	15 (11/4), 67.0 ± 11.0	11 (8/3), 67.0 ± 14.0	LVEF, LVEDV, LVESV
Hastings et al. 2010	6 months	United Kingdom	ASV	NP	Face mask	CHF with SA	SMT	11 (NP), 61.3 ± 10.0	8 (NP), 64.5 ± 8.0	AHI, LVEF, BNP
Johnson et al. 2008	6.9 ± 3.3 months	Canada and United States	CPAP	CPAP: 10.6 ± 1.6 cm H_2_O	Nasal mask Face mask	CHF with OSA	SMT	7 (7/0), 61.0 ± 12.0	5 (5/0), 62.0 ± 9.0	Stroke volume, LVEF, LVEDV, LVESV
Joho et al. 2012	3.5 ± 0.8 months	Japan	ASV	EPAP: 4-5 cm H_2_O IPAP: 3–10 cm H_2_O	Face mask	CHF with CSA	SMT	20 (18/2), 62.0 ± 11.0	12 (10/2), 68.0 ± 9.0	LVEF, LVDd, LVDs, BNP, MSNA
Kaneko et al. 2003	1 month	Canada	CPAP	CPAP: 8.9 ± 0.7 cm H_2_O	NP	CHF with OSA	SMT	12 (11/1), 55.9 ± 2.5	12 (10/2), 55.2 ± 3.6	BP, HR, LVESD, LVEDD, LVEF
Koyama et al. 2010	1 month	Japan	ASV	EPAP: 4 cm H_2_O IPAP: 3–10 cm H_2_O	NP	CHF with SDB	SMT	10 (8/2), 68.4 ± 4.0	7 (4/3), 71.4 ± 7.6	AHI hs-CRP BNP LVEF
Koyama et al. 2011	12 months	Japan	ASV	EPAP: 5 cm H_2_O IPAP: 3–10 cm H_2_O	NP	CHF with SDB	SMT	27 (23/4), 74.8 ± 7.6	16 (13/3), 75.4 ± 6.4	eGFRhs-CRP, LVEF
Mansfield et al. 2004	3 months	Australia	CPAP	CPAP: 8.8 ± 1.4 mm Hg	Nasal mask	CHF with OSA	SMT	28 (28/0), 57.2 ± 1.7	27 (24/3), 57.5 ± 1.6	LVEF, UNE
Naughton et al. 1995	1 month	Canada	NCPAP	CPAP: 10 to 12.5 cm H_2_O	Nasal mask	CHF with CSR-CSA	SMT	12 (NP), 61.0 ± 3.2	12 (NP), 56.6 ± 3.2	LVEF effect of NCPAP on CSA
Oldenburg et al. 2011	12 months	Germany	ASV	EPAP: 4-5 cm H_2_O IPAP: 3–10 cm H_2_O	Face mask	CHF with CSR	SMT and CPAP noncompliance	56 (54/2), 67.7 ± 9.5	59 (52/7), 62.5 ± 11.8	NT-proBNP LVEF parameters of SDB
Pepperell et al. 2003	1 month	United Kingdom	ASV	EPAP: 5 cm H_2_O IPAP: 3–10 cm H_2_O	NP	CHF with CSR	Subtherapeutic ASV	15 (15/0), 71.4 ± 8.6	15 (14/1), 70.9 ± 7.9	Osler test BNP
Tkacova et al. 1997	1 month	Canada	CPAP	CPAP: 10 to 12.5 cm H_2_O	Nasal mask	CHF with CSR-CSA	SMT	9 (NP), 61.0 ± 1.9	8 (NP), 58.6 ± 2.4	LVEF, ANP, MRF
Usui et al. 2005	1 month	Canada	CPAP	CPAP: 7.5 ± 0.5 cm H_2_O	NP	CHF with OSA	SMT	8 (8/0), 55.0 ± 2.0	9 (7/2), 52.2 ± 4.1	MSNA, BP, HR, LVEF
Yoshihisa et al. 2011	6 months	Japan	ASV	EPAP: 4–10 mm Hg IPAP: 4–20 mm Hg	NP	CHF with CSR-CSA	SMT	23 (20/3), 60.8 ± 13.7	37 (29/8), 60.5 ± 16.7	LVEF BNP cardiac systolic and diastolic function

CPAP: continuous positive airway pressure; ASV: adaptive servoventilation; EPAP: expiratory positive airway pressure; IPAP: inspiratory positive airway pressure; NP: not provided; CHF: chronic heart failure; OSA: obstructive sleep apnea; CSR: Cheyne-Stokes respiration; CSA: central sleep apnea; SA: sleep apnea; SDB: sleep-disordered breathing; SMT: standard medical treatment; LVEF: left ventricular ejection fraction; MSNA: muscle sympathetic nerve activity; BNP: B-type natriuretic peptide; AHI: apnea/hypopnea index; eGFR: estimated glomerular filtration rate; hs-CRP: high-sensitivity C- reactive protein; ANP: atrial natriuretic peptide; UNE: urinary norepinephrine; BP: blood pressure; HR: heart rate; MIP: maximal inspiratory pressure; MEP: maximal inspiratory pressure; LVESD: left ventricular end-systolic diameter; LVEDD: left ventricular end-diastolic dimension; LVEDV: left ventricular end-diastolic volume; LVESV: left ventricular end-systolic volume; LVDd: left ventricular end-diastolic dimension; LVDs: left ventricular end-systolic dimension.

**Table 2:** Sensitivity analysis showing the effect sizes for the primary outcomes after removing individual trials included in the meta-analysis.

	LVEF		Overall mortality	
	MD [95% CI]	*P* value	RR [95% CI]	*P* value
All trials	RE: 5.34 [3.85, 6.82]	<0.00001	RE: 1.00 [0.97, 1.03]	0.98
	FE: 3.89 [3.08, 4.69]	<0.00001	FE: 1.00 [0.96, 1.04]	0.95
Naughton et al. 1995 omitted	RE: 5.24 [3.72, 6.75]	<0.00001	RE: 1.00 [0.97, 1.03]	0.98
	FE: 3.83 [3.02, 4.64]	<0.00001	FE: 1.00 [0.96, 1.04]	0.95
Granton et al. 1996 omitted	RE: 5.23 [3.73, 6.73]	<0.00001	RE: 1.00 [0.97, 1.03]	0.98
	FE: 3.84 [3.03, 4.65]	<0.00001	FE: 1.00 [0.96, 1.04]	0.95
Tkacova et al. 1997 omitted	RE: 5.23 [3.72, 6.74]	<0.00001	RE: 1.00 [0.97, 1.03]	0.98
	FE: 3.83 [3.02, 4.64]	<0.00001	FE: 1.00 [0.96, 1.04]	0.95
Kaneko et al. 2003	RE: 5.19 [3.67, 6.72]	<0.00001	RE: 1.00 [0.97, 1.03]	0.98
	FE: 3.75 [2.93, 4.57]	<0.00001	FE: 1.00 [0.96, 1.04]	0.95
Mansfield et al. 2004	RE: 5.45 [3.90, 7.00]	<0.00001	RE: 1.01 [0.97, 1.04]	0.74
	FE: 3.89 [3.08, 4.70]	<0.00001	FE: 1.01 [0.97, 1.05]	0.77
Bradley et al. 2005	RE: 5.63 [4.25, 7.00]	<0.00001	RE: 1.00 [0.97, 1.03]	0.98
	FE: 5.15 [4.14, 6.17]	<0.00001	FE: 1.00 [0.96, 1.03]	0.86
Usui et al. 2005	RE: 5.37 [3.85, 6.89]	<0.00001	RE: 1.00 [0.97, 1.03]	0.98
	FE: 3.89 [3.08, 4.69]	<0.00001	FE: 1.00 [0.96, 1.04]	0.95
Arzt et al. 2005	RE: 5.55 [3.99, 7.12]	<0.00001	RE: 1.00 [0.97, 1.03]	0.98
	FE: 3.94 [3.12, 4.76]	<0.00001	FE: 1.00 [0.96, 1.04]	0.95
Egea et al. 2008	RE: 5.64 [4.04, 7.24]	<0.00001	RE: 1.00 [0.96, 1.03]	0.79
	FE: 4.01 [3.17, 4.85]	<0.00001	FE: 1.00 [0.96, 1.04]	0.96
Johnson et al. 2008	RE: 5.43 [3.86, 7.00]	<0.00001	RE: 1.00 [0.97, 1.03]	0.98
	FE: 3.87 [3.06, 4.69]	<0.00001	FE: 1.00 [0.96, 1.04]	0.95
Ferrier et al. 2008	RE: 5.45 [3.88, 7.01]	<0.00001	RE: 1.00 [0.97, 1.03]	0.98
	FE: 3.88 [3.07, 4.70]	<0.00001	FE: 1.00 [0.96, 1.04]	0.95
Pepperell et al. 2003	RE: 5.50 [3.97, 7.03]	<0.00001	RE: 1.00 [0.97, 1.03]	0.98
	FE: 3.92 [3.12, 4.73]	<0.00001	FE: 1.00 [0.96, 1.04]	0.95
Koyama et al. 2010	RE: 5.04 [3.58, 6.49]	<0.00001	RE: 1.00 [0.97, 1.03]	0.98
	FE: 3.74 [2.92, 4.55]	<0.00001	FE: 1.00 [0.96, 1.04]	0.95
Hastings et al. 2010	RE: 5.09 [3.65, 6.54]	<0.00001	RE: 1.00 [0.97, 1.03]	0.98
	FE: 3.80 [3.00, 4.61]	<0.00001	FE: 1.00 [0.96, 1.04]	0.95
Oldenburg et al. 2011	RE: 5.60 [3.91, 7.29]	<0.00001	RE: 1.00 [0.97, 1.04]	0.79
	FE: 3.90 [3.02, 4.79]	<0.00001	FE: 1.01 [0.96, 1.05]	0.78
Koyama et al. 2011	RE: 5.21 [3.67, 6.75]	<0.00001	RE: 1.00 [0.97, 1.03]	0.98
	FE: 3.70 [2.87, 4.53]	<0.00001	FE: 1.00 [0.96, 1.04]	0.95
Haruki et al. 2011	RE: 5.23 [3.71, 6.74]	<0.00001	RE: 1.00 [0.97, 1.03]	0.98
	FE: 3.82 [3.01, 4.63]	<0.00001	FE: 1.00 [0.96, 1.04]	0.95
Yoshihisa et al. 2011	RE: 5.33 [3.79, 6.88]	<0.00001	RE: 1.00 [0.97, 1.03]	0.86
	FE: 3.84 [3.03, 4.65]	<0.00001	FE: 1.00 [0.96, 1.04]	0.87
Joho et al. 2012	RE: 4.95 [3.52, 6.38]	<0.00001	RE: 1.00 [0.97, 1.03]	0.98
	FE: 3.69 [2.87, 4.50]	<0.00001	FE: 1.00 [0.96, 1.04]	0.95

LVEF: left ventricular ejection fraction; MD: mean difference; RR: risk ratio; RE: random effect model; FE: fixed effect model.

## Abbreviations

NPPV: Noninvasive positive pressure ventilation
cPAP: Continuous positive airway pressure
ASV: Adaptive servoventilation
BiPAP: Bilevel positive airway pressure
SDB: Sleep-disordered breathing
OSA: Obstructive sleep apnea
CSA: Central sleep apnea
CSR: Cheyne-Stokes respiration
LVEF: Left ventricular ejection fraction
LVEDD: Left ventricular end-diastolic dimension
LVESD: Left ventricular end-systolic dimension
BNP: Brain natriuretic peptide
Peak VO_2_: Peak oxygen consumption.
